# High-Throughput Luminescence-Based Serum Bactericidal Assay Optimization and Characterization to Assess Human Sera Functionality Against Multiple *Shigella flexneri* Serotypes

**DOI:** 10.3390/ijms252011123

**Published:** 2024-10-16

**Authors:** Valentina Caradonna, Marika Pinto, Renzo Alfini, Carlo Giannelli, Miren Iturriza, Francesca Micoli, Omar Rossi, Francesca Mancini

**Affiliations:** 1 Laboratory of Molecular Microbiology and Biotechnology, Department of Medical Biotechnologies, University of Siena, 53100 Siena, Italy; v.caradonna@student.unisi.it; 2 GSK Vaccines Institute for Global Health (GVGH), 53100 Siena, Italyrenzo.x.alfini@gsk.com (R.A.); carlo.x.giannelli@gsk.com (C.G.);

**Keywords:** functional assay, SBA, human serum, vaccine, *Shigella*

## Abstract

Shigellosis represents a significant global health concern particularly affecting children under 5 years in low- and middle-income countries (LMICs) and is associated with stunting and antimicrobial resistance. There is a critical need for an effective vaccine offering broad protection against the different *Shigella* serotypes. A correlate of protection has not yet been established but there is a general consensus about the relevant role of anti-O-Antigen-specific IgG and its functionality evaluated by the Serum Bactericidal Assay (SBA). This study aims to characterize a high-throughput luminescence-based SBA (L-SBA) against seven widespread *Shigella* serotypes. The assay was previously developed and characterized for *S. sonnei* and *S. flexneri* 1b, 2a, and 3a and has now been refined and extended to an additional five serotypes (*S. flexneri* 4a, 5b, 6, X, and Y). The characterization of the assay with human sera confirmed the repeatability, intermediate precision, and linearity of the assays; both homologous and heterologous specificity were verified as well; finally, limit of detection and quantification were established for all assays. Moreover, different sources of baby rabbit complement showed to have no impact on L-SBA output. The results obtained confirm the possibility of extending the L-SBA to multiple *Shigella* serotypes, thus enabling analysis of the functional response induced by natural exposure to *Shigella* in epidemiological studies and the ability of candidate vaccines to elicit cross-functional antibodies able to kill a broad panel of prevalent *Shigella* serotypes in a complement-mediated fashion.

## 1. Introduction

*Shigella*, identified in the World Health Organization’s list of antibiotic-resistant “priority pathogens” [1], is a bacterial genus consisting of four serogroups (*S. sonnei*, S. *flexneri*, *S*. *dysenteriae,* and *S. boydii*) and over 50 serotypes [2,3]. This human pathogen gives rise to shigellosis, an acute infection of the small intestine transmitted through the oro-fecal route [4,5] that is a prevalent factor in cases of diarrhea within low- and middle-income countries [6]. Recurrent infections result in impaired cognitive development, stunted growth, and a diminished life expectancy [7,8,9,10]. Shigellosis contributes significantly to global morbidity and mortality, particularly affecting children under 5 years of age [11], with recent data indicating 64,000 deaths in 2016 [12]. No vaccine is yet available against *Shigella,* and the challenge of shigellosis has been further complicated by the emergence of antibiotic-resistant strains [13,14,15,16,17,18,19,20]. The primary serotype accountable for shigellosis in developing nations is *S. flexneri*, with *S. sonnei* being the predominant cause of shigellosis in developed countries [21,22].

The O-Antigen (O-Ag) portion of the membrane lipopolysaccharides of the bacteria determines the differentiation between the different *Shigella* serotypes [23] and is acknowledged as the key antigen for developing protective immunity against shigellosis [24,25,26].

Indeed, the majority of the vaccines against *Shigella* that are currently being developed target the O-Ag. Findings from a Global Enteric Multicenter Study [27] suggested that a vaccine targeting four strains, including the most common serotypes *S. sonnei* and *S. flexneri* 2a, 3a, 6, or 1b, would directly protect against approximately 72% of circulating species and up to 89% when considering cross-protection [28]. Vaccine coverage may increase due to the cross-reactivity between different *Shigella flexneri* strains [29,30]. O-Ag-based *Shigella* vaccine candidates are either in Phase II or initiated Phase III clinical trials [31]. Among them, a four-component formulation based on Generalized Modules of Membrane Antigens (GMMA) has been proposed, targeting *S. sonnei* and *S. flexneri* 1b, 2a, and 3a serotypes [32,33,34], and is currently being tested in a Phase I/II clinical trial (ClinicalTrials.gov number NCT05073003). Stage 1 of the clinical trial, performed in European adults, has been completed, showing the ability of the vaccine to induce good anti-O-Ag-specific IgG bactericidal against the serotypes included in the vaccine [35]. Other multivalent candidate vaccines advanced in clinical development include the quadricomponent *Shigella* bioconjugate vaccine, which carries the O-Ag of *S. flexneri* serotypes 2a, 3a, 6, and *S. sonnei* bioconjugated to the recombinant *Pseudomonas aeruginosa* exoprotein A carrier protein, currently in a Phase II study [36], and the bivalent O-Ag glycoconjugate targeting *S. sonnei* and *S. flexneri* 2a, currently in Phase III [37]. Another candidate vaccine against *Shigella flexneri* 2a is the monovalent carbohydrate-based synthetic conjugate developed by Institut Pasteur, which has demonstrated the ability to elicit a durable immune response in adults [38].

Besides the fact that a definitive correlate of protection against shigellosis has not been established, the measurement of antigen-specific IgG is traditionally used as the primary measure of vaccine immunogenicity [24,26]. Nevertheless, assessing the functionality of antibodies induced by the vaccine in vitro using the Serum Bactericidal Assay (SBA) might serve as an important predictor of efficacy against bacterial pathogens [39,40]. Specifically, concerning *Shigella*, there has been considerable speculation regarding the important role of SBA in providing a more comprehensive characterization of the functional immune response [41,42].

We have developed a high-throughput serum bactericidal assay based on the luminescence readout (L-SBA), which has been already applied to evaluate serum antibody functionality against *S. sonnei* in multiple clinical trials [43,44]. The assay has been also characterized against *S. flexneri* 2a, 1b, and 3a and has been utilized to analyze samples from the first stage of the GMMA four-component vaccine Phase I/II clinical trial, which was conducted in healthy European adults [35]. High bactericidal titers against *S. flexneri* 1b and 3a (numerically higher than those observed against *S. sonnei* and *S. flexneri* 2a) have been observed at baseline in these subjects.

Moreover, in order to assess potential vaccine coverage, it is important to evaluate the vaccine’s capacity to provide broad bactericidal activity against a larger panel of *S. flexneri* serotypes not included in the vaccine [32,45]. Indeed, *S. flexneri* presents an extensive serotype diversity, determined by variation in the O-Ag structure [23], with over 19 known serotypes reported.

In addition to its use in evaluating vaccine coverage, the L-SBA could also be a valuable tool for public health surveillance in Shigella-endemic regions. By enabling the screening of sera for a broad range of Shigella serotypes, the assay could provide crucial insights into the serotype distribution in these populations. This information could then inform the development of targeted vaccination strategies.

In this work, we present the characterization of the L-SBA with human sera to other prevalent *S. flexneri* serotypes (*S. flexneri* 4a, 5b, 6, X, and Y) and the refinement of the assays against different *S. flexneri* 1b and 3a isolates compared to the ones tested previously [46]. The characterization comprises the repeatability, intermediate precision, linearity, specificity, and detection and quantification limits of the assay.

## 2. Results

### 2.1. Setup of L-SBA Conditions on Human Sera Against S. flexneri 4a, 5b, 6, X, and Y Strains

**Figure 1:**
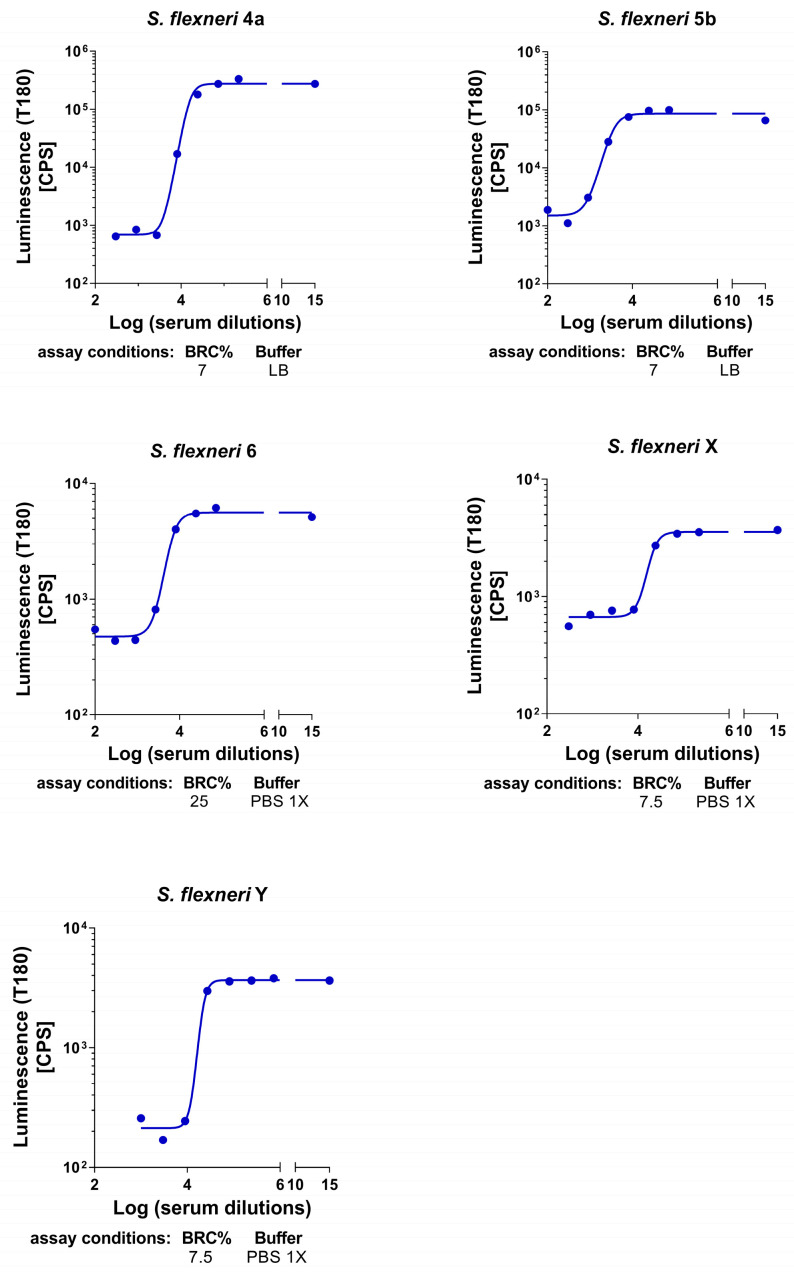
Human standard serum assessed in L-SBA against *S. flexneri* 4a, 5b, 6, X, and Y using the specified assay conditions. 4PL fitting of luminescence at T180 (measured as CPS, counts per second) vs. log-transformed serum dilution. BRC: baby rabbit complement; LB: Luria Bertani medium; PBS: phosphate-buffered saline.

One of the aims of this study was to characterize the L-SBA to evaluate human serum bactericidal activity against *S. flexneri* 4a, 5b, 6, X, and Y strains. The assay conditions, such as the percentage of baby rabbit complement (BRC) and assay buffer established in the preclinical stage for *S. flexneri* 4a, 5b, 6, X, and Y strains [32,45], were applied to L-SBA to test the clinical samples and were found to be optimal for assessing human serum bactericidal activity based on the dose–response curve obtained by probing the human standard serum against each strain (Figure 1).

### 2.2. Optimization of L-SBA on Human Sera Against S. flexneri 1b and 3a

**Figure 2:**
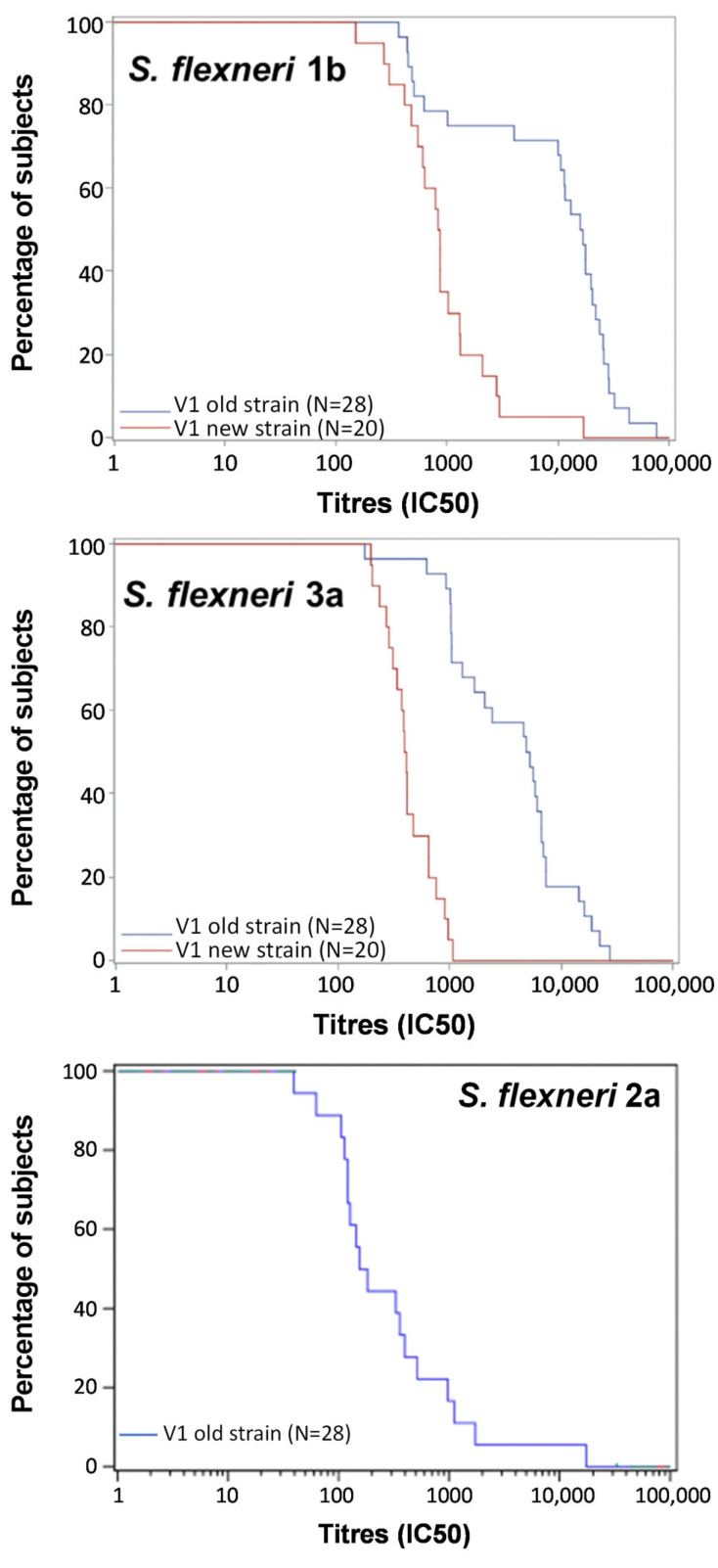
Reverse cumulative distribution plots of SBA titers observed at baseline against the newly retrieved *S. flexneri* 1b NTCT 5 strain and 3a NCTC9989 (new strains) compared to the previously used strains *S. flexneri* 1b NCTC5 Stansfield, *S. flexneri* 3a 6885 and *S. flexneri* 2a strain (old strains). SBA titers have been obtained analyzing sera from individuals enrolled in the NCT05073003 clinical trial. The blue line represents the assay conducted using the old strains, the red line represents the assay conducted using the new strains.

The characterization of the assay against *S. flexneri* 1b and 3a was previously conducted [46] using *S. flexneri* 1b (Stansfield NTCT 5 strain) and *S. flexneri* 3a (6885 strain). However, in the SBA analysis of sera from the NCT05073003 clinical trial, high SBA titers were observed at baseline against *S. flexneri* 1b and 3a strains [35], and this was hypothesized to be due to the sensitivity of the strains used. Consequently, here, alternative *S. flexneri* 1b and 3a strains from Public Health England (PHE) were screened using pooled samples from individuals enrolled in the NCT05073003 clinical trial and collected at baseline (visit 1) or after dose 1 (visit 4). Among the tested *S. flexneri* 1b (NCTC9722, NCTC5, NCTC14120, NCTC8517, and NCTC14154) and *S. flexneri* 3a (NCTC9724, NCTC9989, NCTC7, NCTC9782, and NCTC9783) strains, *S. flexneri* 1b NCTC5 and *S. flexneri* 3a NCTC9989 showed lower SBA titers at baseline. The optimal assay conditions were identified for the two new strains: the selected BRC percentage was 30% for *S. flexneri* 1b and 20% for *S. flexneri* 3a. Baseline titers were further assessed using sera from 20 subjects from the NCT05073003 clinical trial and *S. flexneri* 1b and 3a strains previously used or newly acquired form PHE. Results obtained from running SBA with the *S. flexneri* 1b (Stansfield NTCT 5 strain) and *S. flexneri* 3a (6885 strain) showed that a substantial proportion of subjects had elevated baseline titers (above 5000) against *S. flexneri* 1b (> 70%) and *S. flexneri* 3a (> 50%). In contrast, when the assay was performed using the new strains, less than 20% of tested subjects had elevated baseline titers (above 5000) against *S. flexneri* 1b and no subjects against *S. flexneri* 3a. Baseline SBA titers observed when running the L-SBA with the newly acquired strains were found to be comparable to those observed against *S. flexneri* 2a (Figure 2).

**Figure 3:**
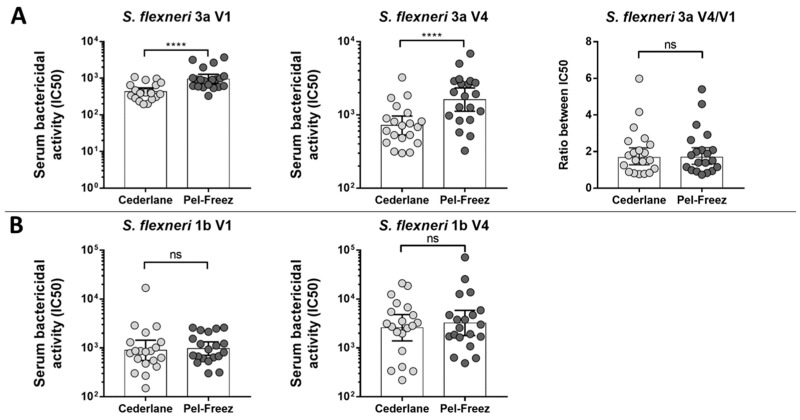
Comparison of bactericidal titers obtained in SBA using Cederlane or Pel-Freez baby rabbit complement for *S. flexneri* 3a (**A**) and *S. flexneri 1b* (**B**) both at visit 1 (V1) and at visit 4 (V4) and comparison between results obtained for samples at the two tested time points (V4/V1). Ns, *p* > 0.05; ****, *p* ≤ 0.0001, Wilcoxon matched-pairs signed rank test.

The same samples were then used to assess the impact of the use of a different source of BRC on L-SBA results. When Pel-Freeze BRC was utilized instead of the BRC obtained from Cederlane used in all SBA experiments conducted so far, no significant differences were observed in IC50 measured at visit 1 (baseline) and visit 4 (post-dose 1) against *S. flexneri* 1b, whereas IC50 values changed for *S. flexneri* 3a but no impact on fold-change over baseline was observed (Figure 3).

### 2.3. L-SBA Characterization Against S. flexneri Serotypes

L-SBA assays performed using the newly selected *S. flexneri* 1b and 3a strains and *S. flexneri* 4a, 5b, 6, X and Y strains where comprehensively characterized.

#### 2.3.1. Precision

**Figure 4:**
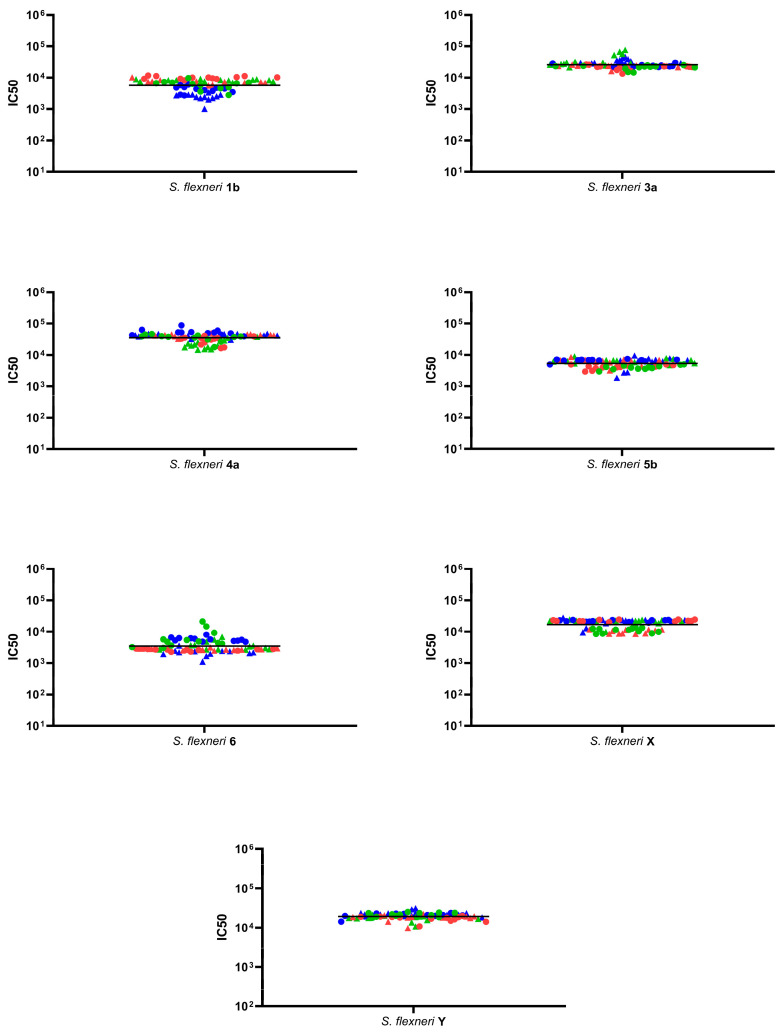
SBA titers (IC50) of standard sera tested in 12 independent replicates. The assay was conducted against the *S. flexneri* 1b, 3a, 4a, 5b, 6, X, and Y strains. Results from individual repeats by each operator are depicted as circles (for operator 1) and triangles (for operator 2), while results obtained for repeats on different days are color-coded as blue (day 1), green (day 2), and red (day 3). The black line represents the geometric mean of all values.

For all tested strains, precision was assessed as intermediate precision (CV% IP) and repeatability (CV% R) testing the same sera multiple times on three different days and by two operators (Figure 4).

The day and the operator, and their interaction, resulted as factors that did not significantly influence the assay’s variability (*p*-values > 0.05). The average LogIC50 from all measurements, standard deviation, and coefficients of variation (CV%) values are reported in Table 1.

#### 2.3.2. Linearity

**Figure 5:**
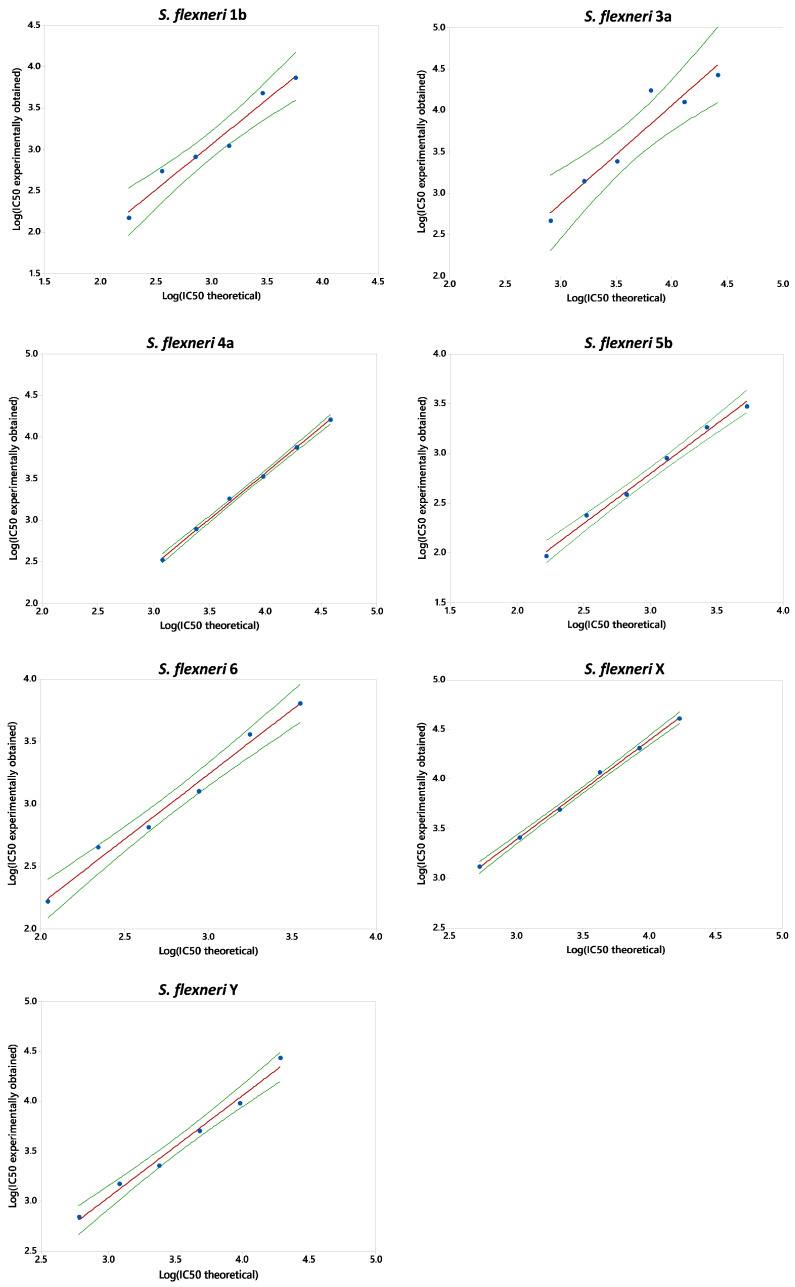
Log(IC50 theoretical) obtained for each sample vs. Log (IC50 experimentally obtained). Single data points are indicated with blue dots. The red solid line represents the linear regression, and the green dashed line represents the regression 95% confidence interval (CI). Data refer to L-SBA conducted against *S. flexneri* 1b, 3a, 4a, 5b, 6, X, and Y, using human standard sera.

In order to assess linearity, the same sample was tested at different serial dilutions. Experimental LogIC50 was plotted against theoretical LogIC50, and a regression analysis was conducted (Figure 5). For each serotype tested, the linear model was significant (*p*-value < 0.05), the intercept (named Constant in Table 2) did not significantly differ from zero (*p*-value > 0.05), and the slope (named T in Table 2) was not significantly different from 1 (95% CI ranges included 1) other than for *S. flexneri* 4a and X. Indeed, only for SBA against *S. flexneri* 4a and X the intercept was significantly different from zero, and the slope for *S. flexneri* 4a was significantly higher than 1. For these two assays, the %error for the IC50 values with respect to the theoretical value was calculated for all tested points and was <20%.

#### 2.3.3. Specificity

To verify that the L-SBA assay was able to assess the functionality of O-Ag-specific antibodies contained in clinical serum against *S. flexneri* 4a, 5b, 6, X, Y, 1b, and 3a, both the homologous and the heterologous specificity of the assay were assessed.

The lowest homologous *S. flexneri* LPS concentration (among the ones tested) that was able to cause the highest IC50 reduction in the IC50 compared to the undepleted control sample was 10 μg/mL for *S. flexneri* 1b, *S. flexneri* 3a, *S. flexneri* 4a, and *S. flexneri* 6, 100 μg/mL for *S. flexneri* 5b and *S. flexneri* X, and 0.5 μg/mL for *S. flexneri* Y. These concentrations were then used to assess the heterologous specificity with the heterologous competitor from the same species (*S. sonnei* LPS) or with the heterologous competitor from a distinct species (*S.* Typhimurium O-Ag).

**Figure 6:**
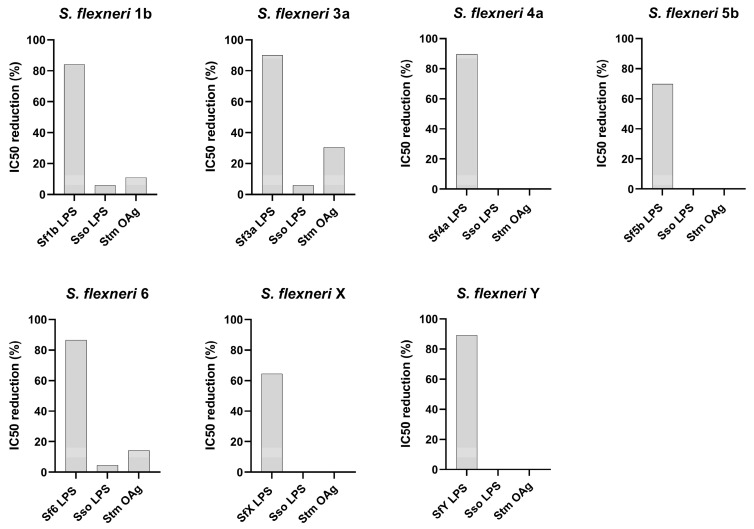
IC50 reduction (%) in L-SBA against *S. flexneri* 1b, 3a, 4a, 5b, 6, X, and Y strains compared to undepleted control samples after incubation with homologous or heterologous competitors. Sf = *S. flexneri*; Sso = *S. sonnei*; Stm = *S*. Typhimurium.

Depletion with the homologous competitor led to an IC50 inhibition of at least 60% in all assays, confirming assay specificity. In contrast, depletion with heterologous antigens caused no decrease or a limited (≤ 30%) decrease in the SBA titer, indicating the absence of any non-*S. flexneri* polysaccharide-specific response in the assay (Figure 6).

#### 2.3.4. Limit of Detection (LoD) and Limit of Quantification (LoQ)

The limit of detection (LoD) of the assay denotes the lowest SBA titer detectable under the assay conditions and the limit of quantification (LoQ) represents the lowest SBA titer that can be quantified with appropriate precision. To create a worst-case scenario for the assay and assess its performance under challenging conditions, the human standard serum was prediluted in order to obtain an IC50 of around 100. This prediluted serum was then assayed in L-SBA against *S. flexneri* 4a, 5b, 6, X, Y, 1b, and 3a serotypes. The results obtained under these conditions are detailed in Table 3.

## 3. Discussion

Addressing the significant disease problem of shigellosis in low- and middle-income countries through the introduction of an effective vaccine that can protect against the most widespread and virulent serotypes of *Shigella* is a pressing priority. For *Shigella*, the anti-O-Ag IgG response has been proposed as a correlate of protection [24,25]. Measuring the functionality of the sera also seems important to better characterize the response elicited by a vaccine candidate or after natural exposure to *Shigella*. The SBA is a widely used in vitro method for assessing serum antibodies’ ability to kill bacteria through complement activation [40,47,48,49], and the luminescence-based high-throughput version of the method has been already developed in order to improve time consumption, cost effectiveness, and labor intensity. This advanced method allows us to evaluate 88 individual sera, excluding controls, per day, per operator, and can be easily adapted to a 384-well plate format to further increase the troughput [50]. This assay has been already used to test serum samples from vaccine clinical trials. This methodology was indeed already characterized in preparation for the assessment of antibody functionality in human sera elicited by a mono-component GMMA-based vaccine against *S. sonnei* [44] and a GMMA-based four-component vaccine against *Shigella* including *S. sonnei* and *S. flexneri* 1b, 2a, and 3a serotypes (ClinicalTrials.gov number NCT05073003) [35]. SBA assays against *S. flexneri* 1b and 3a strains requested further optimization in order to reduce bactericidal titers observed at baseline in European adults. Furthermore, human L-SBA characterization against additional *Shigella* serotypes was necessary in order to evaluate the potential for vaccine cross-protection.

In this study, we described the optimization and intra-laboratory characterization of L-SBA on clinical sera against *S. flexneri* 1b and 3a strains and the intra-laboratory characterization of L-SBA against five additional *Shigella flexneri* serotypes (*S. flexneri* 4a, 5b, 6, X, and Y). Repeatability, intermediate precision, linearity, specificity, LoD, and LoQ were assessed.

SBA against all tested *S. flexneri* serotypes resulted in adequate intermediate precision (CV% IP < 7%) and repeatability (CV% R < 3.9%). The operator and the day and their interaction were confirmed to be non-significant factors in the overall variability. The linearity of the assays was confirmed within the range tested, except for *S. flexneri* 4a and X for which the % error was < 20% at all tested dilutions.

L-SBA also exhibited strong specificity, with ≥ 60% depletion of SBA titers after depletion with homologous LPS and depletion below 30% when depleted with heterologous LPS both from the same *Shigella* species (*S. sonnei* LPS) and from a different bacterial genus, *Salmonella* (S. Typhimurium O-Ag). LoDs and LoQs were also established to understand the lowest SBA titers that can be detected and quantified using the different assays.

## 4. Materials and Methods

### 4.1. Bacterial Strains and Reagents

*S. flexneri* strains were purchased from Public Health England, London, UK. Frozen 20% glycerol stocks of *S. flexneri* strains 1b (NCTC9722, NCTC5, NCTC14120, NCTC8517, and NCTC14154), *S. flexneri* 3a (NCTC9724, NCTC9989, NCTC7, NCTC9782, and NCTC9783), *S. flexneri* 4a (H130920147), *S. flexneri* 5b (H130920151), *S. flexneri* 6 (H130920152), *S. flexneri* X (H130920153), and *S. flexneri* Y (H130920154) were prepared from lyophilized cultures and stored at −80 °C.

The LPS from *S. flexneri* 1b, 3a, 4a, 5b, 6, X, Y, and O-Ag from *Salmonella* Typhimurium were extracted and analyzed for sugar content, protein, and nucleic acid impurities, as previously reported [51].

### 4.2. Serum Samples

The human serum tested to characterize the *S. flexneri* 4a, 5b, 6, X, and Y heterologous strains was obtained by pooling high-responder sera from volunteers originally enrolled in the NCT01229176 clinical study performed in Pakistan and India, which are endemic areas for *Shigella*. High responders were selected based on their anti-*S. flexneri* 1b, 2a, and 3a O-Ag-specific IgG ELISA levels. The frozen 50 µL working aliquots of the serum were stored at −80 °C until use.

A different human serum was used for *S. flexneri* 1b and 3a characterization obtained by pooling high-responder sera from healthy European adults enrolled in the NCT05073003 clinical trial vaccinated with GMMA from *S. sonnei* and *S. flexneri* 1b, 2a, and 3a. High responders were selected based on their anti-*S. sonnei* and *S. flexneri* 2a O-Ag-specific IgG ELISA levels. The frozen 50 µL working aliquots of the serum were stored at −80 °C until use.

All tested samples were heat inactivated (HI) prior to testing in L-SBA at 56 °C for 30 min to remove endogenous complement activity. Various aliquots of HI human standard sera were used and treated as described below to determine the different assay parameters.

### 4.3. Luminescence-Based SBA

The L-SBA was conducted following previously established protocols [46]. Serial dilutions (3-fold dilution steps in 7 dilution points) of the HI sera were made in phosphate-buffered saline (PBS) or Luria Bertani medium (LB) (25 µL/well). After the cultures reached OD600 = 0.22 ± 0.02 (Log-phase), they were diluted to about 1 × 10^6^ Colony Forming Units (CFUs)/mL in PBS. Then, a reaction mixture (75 µL/well) was prepared by combining the previously diluted cultures (10 µL/well), the exogenous BRC (Cederlane or Pel-Freez at different percentages depending on the strain: 30% for the assay with *S. flexneri* 1b, 20% for the assay with *S. flexneri* 3a, 7% for the assay with *S. flexneri* 4a and 5b, 7.5% for the assay with *S. flexneri* X and Y, and 25% for the assay with *S. flexneri* 6), and the assay buffer (LB for *S. flexneri* 1b, 4a, and 5b and PBS for *S. flexneri* 3a, 6, X, and Y). The mixture was finally added to the volume of HI sera dilutions already contained in the wells of the plate to create a final reaction volume of 100 µL. As a control, a row of wells without sera was added to each plate to be sure that the killing was due to the sera and not to the complement (non-specific complement killing). The plates were incubated at 37 °C for 3h. To evaluate bacteria viability at time zero, 5 µL of the bacterial culture diluted in PBS to have about 1 × 10^6^ CFU/mL were added to 45 µL of BacTiter-Glo Reagent (Promega, Southampton, UK) inside a white 96-well plate (Greiner Bio-One, Roma, Italy). After 5 min of incubation on an orbital shaker at 600 rpm in the dark and at RT, the luminescence was detected by a luminometer (Synergy HT, Biotek, Swindon, UK).

After 3 h of incubation, the plate was centrifuged for 10 min at 4000× *g*, and the supernatant was removed. The pellets were resuspended in 100 µL of PBS, and then 50 µL of the suspension was mixed with 50 µL of BacTiter-Glo Reagent (Promega, Southampton, UK) inside a white 96-well plate (Greiner Bio-One, Roma, Italy). After 5 min of incubation on an orbital shaker at 600 rpm in the dark and at RT, the luminescence was detected by a luminometer (Synergy HT, Biotek, Swindon, UK).

### 4.4. Calculations

The assay results are expressed in IC50, with the reciprocal serum dilution causing a 50% reduction in luminescence with respect to the control (indicating 50% growth inhibition). A 4-parameter non-linear regression was applied to the luminescence detected by the luminometer for all tested sera dilutions, and an arbitrary dilution of 10^15^ was assigned to the wells without sera. Since the luminescence levels measured directly correlate with the number of living bacteria in the wells and inversely correlate with the functionality of the antibodies contained in the serum [50], we applied a fitting with a weighting of the data for the inverse of luminescence, constraining the curves to the bottom between 0 and a defined threshold [52]. The threshold was calculated for each serotype tested in the same way: the average luminescence detected in the first 3 dilutions of the tested sera at T180 plus the SD (Standard Deviation) of the luminescence detected. All the values were then rounded to the closest hundred. The plate was validated only if the average luminescence in the wells not containing any sera at T180 was at least 3-fold for *S. flexneri* 1b, 2-fold for *S. flexneri* 3a, 5-fold for *S. flexneri* 4a, 8-fold for *S. flexneri* 5b, 2.5-fold for *S. flexneri* 6, 2-fold for *S. flexneri* X, and 1.5-fold for *S. flexneri* Y regarding the average luminescence at T0.

To be able to calculate an IC50, the highest luminescence detected in the 7 dilutions for each serum had to be at least 0.6-fold that of the luminescence measured in the wells without sera. GraphPad Prism 9 software (GraphPad Software, La Jolla, CA, USA) was used for fitting and IC50 determination.

### 4.5. Precision

The assay precision signifies its capacity to consistently reproduce measurements. Precision was evaluated in terms of repeatability and intermediate precision. Repeatability gauges precision under identical operating conditions and is also called intra-assay variation. On the other hand, intermediate precision measures other kinds of variations—different operators, days, equipment, and calibrants—and it is also known as inter-assay variation.

To assess the precision of the assay, the IC50 for the standard sera against *S. flexneri* 1b, 3a, 4a, 5b, 6, X, and Y was determined independently by two operators, 12 replicates per day, and on three different days (72 measurements in total). HI standard serum obtained by pooling high-responder sera from healthy European adults enrolled in the NCT05073003 clinical trial vaccinated with GMMA from *S. sonnei* and *S. flexneri* 1b, 2a, and 3a was used for the assessment of the precision of the assay against *S. flexneri* 1b and 3a, whereas HI standard serum obtained by pooling high-responder sera from adults from the NCT01229176 clinical study performed in Pakistan and India was used for the assessment of the precision of the assay against *S. flexneri* 4a, 5b, 6, X, and Y. The HI human standard sera were tested starting from 1:100 (final dilution) in L-SBA against *S. flexneri* 5b and 6, from 1:300 (final dilution) in L-SBA against *S. flexneri* 1b and *S. flexneri* X, from 1:500 (final dilution) in L-SBA against *S. flexneri* 4a, and from 1:1000 (final dilution) in L-SBA against *S. flexneri* 3a and *S. flexneri* Y. Log-transformed IC50 results from the three analysis sessions were analyzed with Minitab 18 (Minitab, LLC, State College, PA, USA) applying a mixed-effects model considering the day and operator as random factors to determine the repeatability (CV% R) and intermediate precision (CV% IP) of the assay.

### 4.6. Linearity

HI standard serum obtained by pooling high-responder sera from healthy European adults enrolled in the NCT05073003 clinical trial vaccinated with GMMA from *S. sonnei* and *S. flexneri* 1b, 2a, and 3a was used for the assessment of the linearity of the assay against *S. flexneri* 1b and 3a, whereas HI standard serum obtained by pooling high-responder sera from adults from the NCT01229176 clinical study performed in Pakistan and India was used for the assessment of the linearity of the assay against *S. flexneri* 4a, 5b, 6, X, and Y. The HI human standard sera were diluted in PBS (undiluted, 2-fold, 4-fold, 8-fold, 16-fold, and 32-fold) before being assayed against the *S. flexneri* strains in L-SBA to evaluate the linearity of the assay. A nominal value, referred to as the average IC50 of the undiluted serum, was obtained through precision analysis (Table 1) and served as the expected value against which the theoretical IC50 values, calculated based on the dilutions performed, were compared in linearity analysis. The %error was calculated for all tested points and expressed as LogIC50 theoretical-LogIC50 experimental)/LogIC50 theoretical × 100.

### 4.7. Specificity

Regarding the samples used to assess specificity, to determine the minimum homologous O-Ag concentration able to inhibit IC50 ≥ 70%, HI standard serum obtained by pooling high-responder sera from healthy European adults enrolled in the NCT05073003 clinical trial vaccinated with GMMA from *S. sonnei* and *S. flexneri* 1b, 2a, and 3a was incubated overnight (16–18h) at 4 °C via shaking in an orbital shaker at 200 rpm with an equal volume (1:1; *v*:*v*) of the homologous competitor at the final concentrations of 100, 50, 10, 2, and 0.5 µg/mL or PBS alone prior to being assayed in SBA against each *Shigella* serotype.

The lowest concentration of LPS between the ones tested able to inhibit the IC50 > 70% was then used in a second experiment to determine the heterologous specificity. To determine the heterologous specificity, *S. sonnei* LPS (heterologous but from the same species) and *Salmonella* Typhimurium OA-g (heterologous but from a different species) were used as inhibitors. Internal controls for this experiment were represented by a sample preincubated overnight at 4 °C via shaking at 200 rpm with an equal volume of PBS (undepleted) and a sample preincubated with homologous *S. flexneri* O-Ag (homologous). Each spiked sample was assayed on the same day.

Specificity was determined as % IC50 inhibition, calculated using the following formula:

$$\%IC50\;inhibition\;=\;\frac{\left(IC50\;of\;the\;undepleted\;sample)-(IC50\;of\;the\;depleted\;sample\right)}{\left(IC50\;of\;the\;undepleted\;sample\right)}\times 100$$

### 4.8. Limit of Detection and Limit of Quantification Assessment

HI standard serum obtained by pooling high-responder sera from healthy European adults enrolled in the NCT05073003 clinical trial vaccinated with GMMA from *S. sonnei* and *S. flexneri* 1b, 2a, and 3a was used for the assessment of the LoD and LoQ of the assay against *S. flexneri* 1b and 3a, whereas HI standard serum obtained by pooling high-responder sera from adults from the NCT01229176 clinical study performed in Pakistan and India was used for the assessment of the LoD and LoQ of the assay against *S. flexneri* 4a, 5b, 6, X, and Y. HI standard human sera were diluted v:v in PBS 60 times prior to being assayed in SBA against *S. flexneri* 1b, 70 times prior to being assayed in SBA against *S. flexneri* 3a, 400 times prior to being assayed in SBA against *S. flexneri* 4a, 50 times prior to being assayed in SBA against *S. flexneri* 5b, *S. flexneri* 6, and *S. flexneri* Y, and 90 times prior to being assayed in SBA against *S. flexneri* X to obtain an IC50 of around 100. Twelve identical diluted samples were assayed on the same day by the same operator starting with a 1:4 dilution.

The LoD and LoQ of the assay were calculated according to the ICH guideline Q2(R2) [53] by applying the following formulas:

$$LoD=10^{\left(3.3\times SD\right)}\times X$$

$$LoQ=10^{\left(10\times SD\right)}\times X$$

where X represents the lowest serum dilution tested in the assay (e.g., 4) and SD represents the standard deviation of IC50 obtained for the samples.

### 4.9. Statistical Analysis

Statistical analyses were conducted using Minitab 18 (Minitab Inc., Chicago, IL, USA). Variance component analysis using a mixed-effect model with Restricted Maximum Likelihood (REML) was employed to assess the intermediate precision (defined as the variability among different days and different operators), the repeatability (defined as the variability under the same operating conditions over a short interval of time), and the contributions of the day of analysis and operator to the variability. Regression analysis was utilized to evaluate linearity.

## 5. Conclusions

L-SBA has already been characterized as able to obtain good performance against other bacterial pathogens using both animal and human samples [46,52]. Here, the assay was characterized against a broad panel of *Shigella* serotypes, confirming that it can be easily extended to other strains. Such an assay is relatively rapid and cost effective and is being used for the analysis of samples coming from Phase I/II *Shigella* clinical trials. Additional work is planned to further qualify the assay and use it to analyze sera from a Phase III trial, further supporting the development of a vaccine against *Shigella*.

## Figures and Tables

**Table 1:** Assay precision. Number of observations (n), average LogIC50, standard deviation (SD), CV% day, CV% operator, CV% day*operator, CV% IP, and CV% R obtained in L-SBA against *S. flexneri* 4a, 5b, 6, X, and Y strains.

	LogIC50						
	*S. flexneri* 1b	*S. flexneri* 3a	*S. flexneri* 4a	*S. flexneri* 5b	*S. flexneri* 6	*S. flexneri* X	*S. flexneri* Y
n	72	72	72	72	72	72	72
Average	3.7	4.4	4.5	3.7	3.5	4.2	4.2
SD	0.2	0.1	0.2	0.1	0.2	0.1	0.0
CV% day (*p*-value)	5.8 (0.191)	0.7 (0.418)	4.0 (0.181)	0.0 (0)	0.0 (0)	4.3 (0.16)	1.0 (0.253)
CV% operator (*p*-value)	0.0 (0)	2.0 (0.289)	0.0 (0)	1.0 (0.409)	3.1 (0.341)	0.4 (0.313)	0.0 (0)
CV% day*operator (*p*-value)	3.0 (0.125)	1.6 (0.192)	1.6 (0.153)	2.4 (0.107)	4.4 (0.091)	0.0 (0)	0.8 (0.181)
CV% R	2.6	2.2	2.4	3	3.8	1.6	1.7
CV% IP	7	3.4	4.9	4	6.6	4.6	2.1

**Table 2:** Coefficients of the regression analysis for assay linearity evaluation of each of the *S. flexneri* serotypes tested. The Constant term represents the intercept of the regression line whereas the T term represents the slope.

	Term	Coef	95% CI	*p*-Value
*S. flexneri* 1b	Constant	−0.205	(−1.168; 0.759)	0.587
	T	1.088	(0.772; 1.404)	
*S. flexneri* 3a	Constant	−0.681	(−2.531; 1.170)	0.365
	T	1.185	(0.684: 1.686)	
*S. flexneri* 4a	Constant	−0.857	(−1.0971; −0.6172)	0.001
	T	1.1057	(1.0436; 1.1678)	
*S. flexneri* 5b	Constant	−0.208	(−0.577; 0.162)	0.194
	T	1.0005	(0.8780; 1.1229)	
*S. flexneri* 6	Constant	0.128	(−0.350; 0.605)	0.499
	T	1.036	(0.8678; 1.2041)	
*S. flexneri* X	Constant	0.3808	(0.1580; 0.6037)	0.009
	T	1.002	(0.9386; 1.0654)	
*S. flexneri* Y	Constant	−0.018	(−0.589; 0.552)	0.933
	T	1.0178	(0.8579; 1.1777)	

**Table 3:** LoD and LoQ (IC50) of the assay assessed by testing prediluted standard serum against each of the tested *S. flexneri* strains. Values have been calculated following the ICH guidelines Q2(R2) as detailed in Section 4.8.

	*S. flexneri* 1b	*S. flexneri* 3a	*S. flexneri* 4a	*S. flexneri* 5b	*S. flexneri* 6	*S. flexneri* X	*S. flexneri* Y
LoD	9	7	5	7	7	10	6
LoQ	42	21	8	26	18	68	14

## Data Availability

Data are contained within the article. The raw data supporting the conclusions of this article will be made available by the authors upon reasonable request.
